# Review: New sensors and data-driven approaches—A path to next generation phenomics^☆^

**DOI:** 10.1016/j.plantsci.2019.01.011

**Published:** 2019-05

**Authors:** Thomas Roitsch, Llorenç Cabrera-Bosquet, Antoine Fournier, Kioumars Ghamkhar, José Jiménez-Berni, Francisco Pinto, Eric S. Ober

**Affiliations:** a Department of Plant and Environmental Sciences, University of Copenhagen, Thorvaldsensvej 40, 1871 Frederiksberg C, Denmark; b LEPSE, INRA, Montpellier SupAgro, Univ Montpellier, Montpellier, France; c Arvalis, Institut du végétal, 45, voie Romaine 41240 Beauce la Romaine, France; d Forage Science, Grasslands Research Centre, AgResearch, Tennent Drive, Fitzherbert, Palmerston North 4410, New Zealand; e Instituto de Agricultura Sostenible, Consejo Superior de Investigaciones Cientificas (CSIC) Avenida Menéndez Pidal, Campus Alameda del Obispo, 14004 Córdoba, Spain; f Global Wheat Program, International Maize and Wheat Improvement Center (CIMMYT), El Batán, Texcoco, México C.P. 56237, Mexico; g National Institute of Agricultural Botany (NIAB), Huntingdon Road, Cambridge, CB3 0LE, UK; h Department of Adaptive Biotechnologies, Global Change Research Institute, CAS, Brno, Czech Republic

**Keywords:** CGIAR, consultative group for international agricultural research, CMOS, complementary metal-oxide-semiconductor, DIY, do it yourself, EMI, electromagnetic induction, FAIR, findable, accessible, interoperable and reusable, HTP, high-throughput phenotyping, IPPN, international plant phenotyping network, IR, infrared, LiDAR, light detection and ranging, LIFT, laser-induced fluorescence transient, MIAPPE, minimum information about a plant phenotyping experiments, MWIR, mid-wavelength infrared, NDVI, normalised difference vegetation index, NIR, snear-infrared spectroscopy, RGB, red-green-blue, RUE, radiation use efficiency, SIF, sun-induced fluorescence, SWIR, short-wavelength infrared, UAV, unmanned aerial vehicle, WUE, water use efficiency, Imaging, IPPN, Metadata, Next generation phenomics, Plant phenotyping, Sensor development, Trait value

## Abstract

At the 4th International Plant Phenotyping Symposium meeting of the International Plant Phenotyping Network (IPPN) in 2016 at CIMMYT in Mexico, a workshop was convened to consider ways forward with sensors for phenotyping. The increasing number of field applications provides new challenges and requires specialised solutions. There are many traits vital to plant growth and development that demand phenotyping approaches that are still at early stages of development or elude current capabilities. Further, there is growing interest in low-cost sensor solutions, and mobile platforms that can be transported to the experiments, rather than the experiment coming to the platform. Various types of sensors are required to address diverse needs with respect to targets, precision and ease of operation and readout. Converting data into knowledge, and ensuring that those data (and the appropriate metadata) are stored in such a way that they will be sensible and available to others now and for future analysis is also vital. Here we are proposing mechanisms for “next generation phenomics” based on our learning in the past decade, current practice and discussions at the IPPN Symposium, to encourage further thinking and collaboration by plant scientists, physicists and engineering experts.

## Highlights

• Strategies for future high throughput, non-destructive and cost-efficient measurement of plant traits are highlighted.
• Use of low-cost and DIY approaches in phenomics provides opportunities for rapid prototyping and sensor development.
• Robust protocols, data harmonization and provenance are critical to allow data reuse and cross validation of phenotypes.
• Below-ground phenotyping is a major bottleneck and new technologies allowing the measurement of root-related traits are needed.

## 1 Introduction

Advances in the ability to quantify the expression of traits on large numbers of plants has exceeded expectations in the past decade or two. However, the greater decrease in the cost of genotyping has maintained phenotyping as the major bottleneck for gene discovery and molecular marker development. Recent progress in phenotyping has been thoroughly reviewed [[1], [2], [3], [4], [5], [6]] and the number of papers published describing innovations in sensor technologies and phenotyping methods has increased steadily (see citations here and in recent reviews, above). At the 4^th^ International Plant Phenotyping Symposium meeting of the International Plant Phenotyping Network in 2016 at CIMMYT in Mexico [7], a workshop was convened to consider challenges and opportunities, and to explore the way forward with sensors for phenotyping. This paper discusses some of the points arising from that workshop, but is not a comprehensive treatment of all the current issues in this area. The objective here is to highlight a few important matters in the phenotyping community, to initiate new thinking and research activity and issue a call for joint community effort across disciplines towards next generation phenomics. In addition, a brief review of the phenotyping horizon and new-generation sensors provides a glimpse of new tools that may be available in the near future.

Sensor-based plant phenotyping is an essential and integral part of a holistic phenomics approach to address the complex genotype x environment x management (GxExM) interactions in fundamental and applied plant science research, germplasm screening in breeding programs, precision agriculture and digital farming. Phenomics can be integrated into a systems biology multi-omics approach [8]. Whereas most non-invasive methods have been originally developed for high-throughput phenotyping (HTP) applications under controlled conditions, the increasing number of field applications provides new challenges and often requires specialised solutions.

**Fig. 1:**
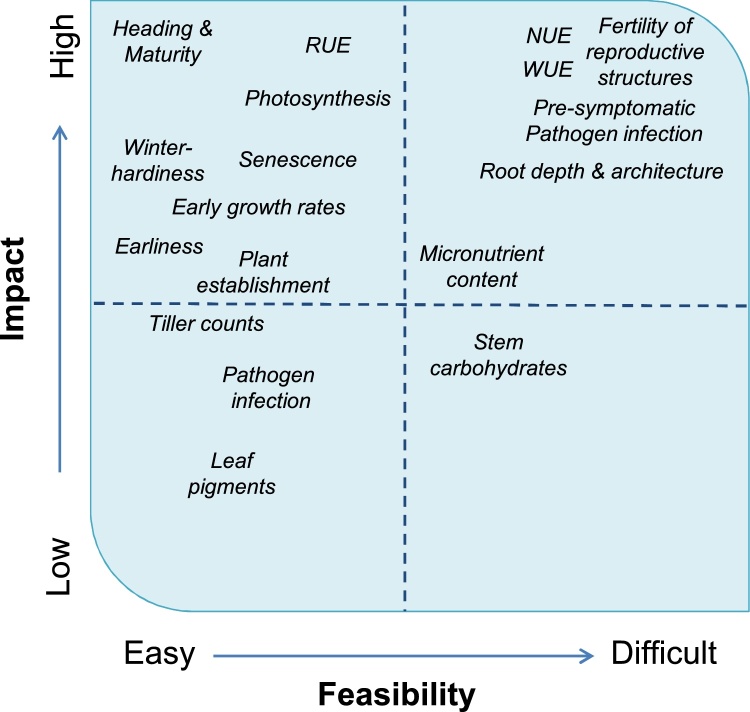
Impact vs feasibility analysis for the estimation of agronomic traits by sensor and imaging technologies. See List of Abbreviations.

There are extensive lists of key phenotypes that must be measured to understand specific questions of plant function, or target traits that contribute to improvements in crop yield, yield stability, resource capture and use efficiency (e.g. water and nitrogen), quality (or chemical composition) of the harvested product, or resistance to abiotic or biotic stresses. Furthermore, various mechanisms contribute to multi-genic traits, so the individual underlying trait components must be phenotyped. Many plants also exhibit important characters that are unique to their species; in brief, there is no shortage of phenotyping challenges. Some well-established stories have been around for more than half a century, such as assessing vegetation vs. non-vegetation via remote-sensing and using multispectral sensors to quantify the dynamics of canopy growth and senescence [9]. The emphasis has been rightly placed, as the extent of light capture and utilization by the canopy drives productivity. However, there are many other traits that are vital to plant growth and development, often requiring measurement at the appropriate temporal and spatial scales. These demand HTP phenotyping approaches that are still at early stages of development, or elude current capabilities. Examples, discussed further below, include fertility of reproductive structures; photosynthetic rate; biomass; growth, water and nutrient uptake activity of roots in the field, etc. Thus, complex or mega-traits need to be broken down into component traits that can be monitored and quantitatively assessed using the appropriate choice of sensor. Table 1 contains a compilation of various agronomically relevant traits related to growth and development, morphology, physiology, biotic interactions, and the relevant tissue that need to be phenotyped. The currently used methods and their limitations are complemented by the technologies under development, which are elaborated in Section 2. The feasibility and impact of these traits on plant biology and crop improvement varies considerably (Fig.1). The constraints and demands of current and prospective phenotyping solutions, such as development costs and time, need to be taken into account to prioritise the focus for improvements, which is addressed in Section 3.

**Table 1:** Current challenges for the determination of some agronomically relevant crop traits by sensor-based techniques and technical solutions under development. *See "Technology Readiness Levels (TRLs) in the Project Lifecycle" http://tinyurl.com/y7gbf28c.

Target Trait	Scale	Current limitations	Current method	Technologies under development (TRL)*
**1. Growth, morphology**				
Heading and maturity	Plant	Resolution; accurate feature detection	Visual scoring	Cereal spike counts from images (7)
Winter hardiness, plant establishment	Plant/plot	Image pre-processing and automated analysis	Visual counting	Plant counts from images (7)
Biomass	Plant, canopy	Estimation of bio-volume vs actual weight	Fresh and oven dry weight	LIDAR (5) SWIR (5)
Lodging	Plant	Subjective	Visual scoring	Video imaging to measure plant oscillation (5); ultrasonic distance sensors (5); force transducer (6)
Root development	Plant	slow, laborious manual methods	Soil coring; excavations; rhizotrons (controlled environment)	Ground penetrating radar (4); Electromagnetic induction(4); Tomographic root imaging

**2. Physiology**				
Water use efficiency	Plant, canopy	Measurement of water use and biomass slow, often only indirect estimations; scaling from tissue to crop	Destructive and gravimetric; estimation via C and O isotopic ratios	LWIR, NIR (7); Thermal imaging (7); Fusion of chlorophyll fluorescence and thermal imaging (6)
Photosynthesis, transpiration	Leaf, plant, canopy	Upscaling, model specificity	Gas exchange; estimation via fluorescence at low O_2_, O isotopic ratio	Sun-induced chlorophyll fluorescence (6); LIFT (6)
Leaf water status	Leaf	Slow, destructive, Low precision	gravimetric, psychrometry	Leaf clip SWIR (4); THz sensing
Nitrogen uptake efficiency	Plant	Indirect estimation of N	Isotopic tracer ^15^N tracers	Hyperspectral imaging for N concentration (6)
Shoot Nitrogen content	Plant	Indirect estimation of N (chlorophyll as surrogate), not accounting for grain N	Destructive and wet chemical analysis	Estimation via multi-spectral LiDAR (5); Hyperspectral imaging
Stem carbohydrates	Stem	Assays slow; cannot resolve fructan species; low precision via NIR	Colorimetric assays; HPLC, NIRS	Hyperspectal detection (5)
Grain protein content	Grain	Specificity; application of harvested grain, not proven on intact organs	NIRS, wet chemistry	Hyperspectral sensing (6)

**3. Biotic Interactions**				
Pathogen infection	Organ	Sensitivity, specificity at the level of species/pathotype	Visual scoring, multispectral; computer vision	Hyperspectral imaging (4);
Pre-symptomatic detection of pathogens	Organ	Sensitivity, specificity	Immuno- or DNA/RNA-based methods	Hyperspectral (4); Fluorescence (4); Thermography (4)
Weed detection	Plant, canopy	Resolution; accurate feature detection; speed	Computer vision	Hyperspectral imaging (4); image feature recognition(5)

**4. Development**				
Growth stage determination	Plant	Slow	Manual; some dissection to visualize internal structures	In-field x-ray tomography (4)
Tuber development	Plant	Slow	Destructive harvest	In-field x-ray tomography (4)
Senescence	Plant	Specificity, sensitivity	Visual scoring	Hyperspectral imaging (5); LiDAR (red light) (5)

Sophisticated instrumentation and platforms are being developed to increase throughput (the numbers of plants/genotypes that can be phenotyped per experiment or per year). However, in many cases costs also increase, putting many of these achievements out of reach for researchers or companies with limited budgets. Static or plant-to-sensor facilities (indoor or field-based) also have limited capacity, and only a fixed number of projects can be taken into the pipeline. Therefore, there is growing interest in low-cost sensor solutions, and mobile platforms that can be transported to the plants, rather than the plant to the platform. Also, user needs in throughput, cost-efficiency, precision, spatial and temporal resolution, accuracy and precision, user friendliness, degree of automatization and complexity of data output are very diverse. Thus, various types of sensors are required to address the very diverse and objective-bound needs. The number of commercially available sensors provided by the industry are currently limited and not able to cover all the diverse and specialised needs of scientists and end users. Areas of challenge in coming years are to: scale-up low throughput methods; scale-down large or heavy equipment; reduce the cost of expensive instruments without extensively compromising precision or reliability; accelerate methods by narrowing down to the essentials. Examples of recent developments in low-cost and ‘do-it-yourself’ (DIY) sensors, and the requisite cautions, are discussed further, below.

One of the hurdles for next generation phenomics is to collect the right data on the right target at the right time and under the right conditions to address the right questions. Other components are the essential step of converting these data into knowledge, and ensuring that these data (and the appropriate metadata) are stored in such a way that they will be intuitive and available to others now and for future analysis.

## 2 Next-generation sensors

Instrumentation capabilities are in constant evolution toward greater scales and throughput, aiming to describe more and more complex phenomena. Initially, adapting off-the-shelf technologies for phenotyping applications has been the main trend. More recently, greater sophistication in adapting acquisition solutions have appeared. Nowadays, the increased visibility of the HTP domain, coupled with strong technological investment from scientific teams, is driving sensor manufacturers to adapt their products to specific uses for measurements of plant traits. For example, industrial Light Detection and Ranging (LiDAR) systems working in the red band can be used for spatial distribution of green tissues within a canopy [10]. LiDAR provides a detailed 3D reconstruction of the canopy but lacks information about the canopy bulk density, which is necessary for estimating actual biomass [11]. The estimation of actual biomass could be enhance by several approaches: combining the 3D plant reconstruction from LiDAR, aerial imagery, and spectral [[12], [13], [14]] or microwave sensing [15]; RGB imaging followed by a void filling process, or adjusting the contrasts between dark and light colours [16]. Such fusion of LiDAR and multi-spectral imaging in one sensor, which would allow simultaneous retrieval of structural and biochemical traits without the limitations of passive remote sensing, has been proposed at different conceptual levels [[17], [18], [19], [20], [21]]. This is now available for aerial vegetation mapping and land cover classification [22,23]. However, similar modalities for mobile, ground-based imaging are still limited. Such a system would accelerate and improve the precision and accuracy of field phenotyping enabling applications where the vertical distribution of photosynthetic pigments or nitrogen across the canopy could be estimated. Phenospex (The Netherlands) has recently released a gantry-type [24] multispectral LiDAR for phenotyping applications (model PlantEye F500). With simultaneous 4 spectral channels in 400–900 nm and 3D point clouds, this will enable new trait discovery in that direction.

Also, methods based on chlorophyll fluorescence are advancing, such as relatively inexpensive hand-held instruments designed for collaborative field research (https://photosynQ.org/, Michigan State University, USA); or Multiplex – Force-A, France) [25], or Laser-Induced Fluorescence Transient (LIFT) [26,27] and sun-induced fluorescence (SIF) methods [28] that target canopies in the field.

Hyperspectral imaging is rapidly advancing with new high-resolution cameras and scanners that cover visible and near infrared (VNIR 400-1.000 nm), short wave infrared (SWIR: 1.000–2.500 nm) and beyond (e.g. long wave infrared LWIR: 8–15 μm). VNIR hyperspectral cameras, in particular, are becoming miniaturized and therefore suitable to be mounted on UAVs for phenotyping applications. LWIR cameras for infrared thermometry are becoming quite common in phenotyping both for manned [29] and unmanned operations [30]. However, given the cost and complexity of hyperspectral LWIR cameras, most commercial systems are currently based on a single-broadband camera based on uncooled detectors (microbolometers). SWIR on the other hand, are becoming an option but still are bulkier and more expensive than their VNIR counterpart; therefore, their operation is restricted to manned vehicles [31]. These sensors have to be calibrated by screening genotypes at the same growth stages and in different environments to detect the slightest variabilities, and there are particular challenges for obtaining good data from measurements using aerial [32] or ground vehicles [33]. Furthermore, the time of day and other factors affecting light intensity and quality should be considered during calibration because spectro-radiometric cameras and scanners use natural light conditions or external light sources [34]. These powerful cameras and scanners can be useful tools for multi-trait screening, specifically monitoring traits under abiotic stress, such as early signs of chlorosis before it is detectable by RGB cameras, or plant water status [29,35].

Despite the potential of hyperspectral cameras for estimating biochemical composition of plants, its applicability to sensing elements and micro-elements is very limited given that they do not have specific spectral absorption features. UV fluorescence emission from a material could lead to the development of next generation sensitive and inexpensive fluorescence scanners [36] for phenotyping concentration of elements in plants. Through correct design, integrated sensors with capacity of strong fluorescence capture are on their way, which may be very useful for high-throughput field screening of nutritional components and micro-elements. Graphene-based plasmonic nano-antenna arrays have been proposed, designed and tested for fluorescence sensing [37]. It is clear that among all these emerging technologies, there is no universal solution in the horizon, and it is likely to be the synergistic combination of different sensor technologies what could overcome some of the limitations of specific technologies used in isolation.

Imaging and sensor technologies for future field phenotyping must be designed to incorporate metadata from the experiment and at least include plant ID, plot identification, plant water status, soil surface correction, soil surface temperature, soil surface water content (see Section 5). We should also aim to deliver inexpensive, user-friendly, robust and fast solutions where that metadata is linked to the sensor data and turned into traits in an automatic or semi-automatic way. To develop these tools, the user must be able to also manually change the settings for any real-world scenario, which may cause errors in the automation. Some examples exist in the literature where pipelines have been described for processing field phenomic data for RGB imagery [[38], [39], [40]] thermal [29], LiDAR [11,41,42], and UAV data [43,44].

In-field plot segmentation and real time data processing, quantification and analysis is critical for end-users and is now partially practical [41] and operational with combination of modalities such as LiDAR and visible (RGB) imaging. Although use of LiDAR is a well-established method for estimation of plant biophysical traits [41,[45], [46], [47]], and detailed characterization of plants in the field [11,12,[48], [49], [50], [51]], the real time data processing and analysis with automated and error free plot detection is not universally available yet. Initial efforts are very promising [52] but needs support from the farming, seed and breeding industries as well as technology development companies to streamline this approach in any future field-based phenotyping. During the workshop and throughout the symposium a strong demand for below-ground phenotyping of root traits became evident. Underground traits are notoriously more difficult to measure than shoot traits, but x-ray microcomputed tomography of roots in soil-filled pipes has opened up new possibilities for non-invasive imaging [53,54]. In the field, non-invasive estimation of root activity is possible via soil drying profiles computed using electromagnetic induction or electrical resistance tomography methods [55], although computational complexities require further software development before EMI is realized as an HTP method. There is potential for further breakthroughs in root phenotyping instrumentation to have huge impact on breeding and crop improvement.

Imaging plant components within tissues or soils (‘see-through technologies’) is a field that currently needs further development and focus. Ground penetrating radar provides vision for sub-surface parts of the plants (roots and tubers) by capturing reflection, refraction, and scattering of high-frequency radio waves [56,57] with its antennae within a certain distance from the soil. Further, terahertz (THz) imaging is a progressing technology that detects water content of plant tissues with some promising early results in lab experiments [[58], [59], [60], [61]]. However, its penetration power is extremely low and its estimates are not consistent with field screening data. To improve these methods, lasers and nano-plasmonic light concentrators [58] are being combined with this technology, which may result in more penetrating power, and hence more resolution when imaging shoots or roots, at least initially in the lab. Recent advances have made non-destructive evaluation of ceramic objects possible using this technology [62]. This may lead to the use of THz technology for scanning roots through dry soil. Another emerging see-through technology is Wi-Fi holography, where stray Wi-Fi radiation can be used to construct 3D holographic images of meter-sized objects within buildings [63]. Plant scientists can be early adopters of these technologies by communicating problems in phenotyping to developers working the fields of physics and engineering.

Engineers and manufacturers often encounter difficulties in assessing the global market for new phenotyping technology and finding fit for purpose data analytics used experimentally by researchers. Spin-off ventures from academic research can sometimes help spread market opportunity into other, larger sectors, such as precision agriculture. UAVs equipped with multispectral cameras are now operated routinely for generating vegetation index maps that, at least in principle, could assist agronomists and farm managers to optimize decisions such as how much fertilizer should be applied and where. Other technologies such as sensor networks or thermal and hyperspectral imaging for monitoring crop water stress have also been demonstrated [[64], [65], [66]]. However, the reality is that these technologies seem to be far from practical application, and the potential economic success of any one technology is difficult to predict. In that regard, phenomics can help to close the gap between ‘pretty pictures’ and plant physiology, providing robust yet user-friendly applications of sensor technologies. in precision farming. The same algorithms that are currently used to screen physiological traits in breeding trials could be translated to the farm scale to provide maps of actual crop status, which could be a useful management tool for agronomists. Development of new technologies that would benefit the phenotyping and whole plant science communities can be facilitated by increasing communication of needs and solutions between sensor designers, programmers, and researchers across disciplines: plant biology, photonics, physics, engineering, computer science, mathematics, etc. A key challenge is that the information desired by the academic sector can be complex, which may not be needed by breeding programs, and becomes an obstacle in precision agriculture. Typically, a simple yes or no answer is required by farmers for many farm operations (e.g. is the crop at the correct growth stage for fungicide application?). For more detailed questions of where and how much, automatized variable rate technologies for application of fertilizers, growth regulators or pesticides, simple and robust solutions derived from some level of crop intelligence are required to implement it them. Making such connections between phenotypic data and practical applications will enrich the technological offer for academia and industry.

## 3 Low-cost or DIY phenotyping solutions

Cutting-edge, disruptive technologies have great potential to revolutionize phenotyping capabilities. However, they often come at a cost: technology that has not yet been widely commercialised is usually expensive, as production is limited to a small number of units, or it is limited to bespoke construction for individual users. Depending on the requirements of the experiment, there can be low-cost alternatives to high-end, high resolution instruments, such as using laser scanners built for the gaming industry to measure crop architecture features [67], and ultrasonic distance sensors for crop height [68], when LiDAR is not affordable or its precision is not required.

With the advent of open source software and hardware initiatives, some disruptive projects emerged that have enabled a democratization of sensor development, reducing the entry barrier in terms of cost and expertise. Platforms such as Arduino (https://www.arduino.cc) or Raspberry Pi (https://www.raspberrypi.org), together with strong associated communities provide the building blocks for very rapid prototyping of sensor technologies that have been fostered by the scientific and academic communities [38,69]. This is the ethos behind organizations such as Public Lab (https://publiclab.org/), which host methods describing DIY spectrometers, thermal cameras and multispectral sensors using readily available materials.

The spectrometer (v 3.0) claims 3 nm wavebands from 400 to 700 nm, built using a webcam (e.g. Gumstix, Inc., USA) and a DVD for a diffraction grating and signals processed using open source software (https://spectralworkbench.org/). The DIY Plant Analysis Kit ($10, Public Lab; Infragram.org) helps users convert a digital camera into a multispectral camera by replacing the IR filter and adding a theatre gel blue filter so that the camera’s red channel registers mostly near infrared. A similar attempt has been the construction of multi-spectral cameras using Raspberry Pi components and 3D printers for the housing (http://www.khufkens.com/projects/tetrapi/).

However, the fast adoption of these DIY approaches and novel technologies also poses some risks in terms of data quality, the robustness of the data produced and data interpretation [70]. For example, the Plant Analysis kit (Infragram.org) claims that the user can ‘measure photosynthesis’; most plant scientists will appreciate that estimating ‘greenness’ does not necessarily equate to photosynthetic rate. NDVI was formulated as the normalized ratio of red and NIR reflectance. In most cases of converted cameras, the red channel is blocked so it can be used as the source of NIR, replacing the red band in the calculation of the NDVI by the blue or green bands. The quantum efficiency of CMOS sensors in consumer cameras in the IR bands is generally low, though solutions may be on the horizon [71]. Therefore, as the use of DIY multispectral cameras is becoming increasingly popular and NDVI maps and figures from these cameras installed in UAVs are published online, the information about the specifications of the cameras are often missed or vaguely described. Similarly, the miniaturisation and cost reduction of thermal cameras are providing popular solutions for mounting these onboard UAVs. Again, the need for sensor calibration to provide actual temperature values becomes critical when the application goes beyond pretty pictures and the aim is to produce robust quantitative data on the physiological traits of interest.

Another generic problem with low-cost or DIY sensors is that the provenance and quality of components differs with each unit, making it difficult to share and compare data, unless sensors are calibrated according to an internationally accepted standard. Most commercial manufacturers would adhere to such standards, so as more DIY sensors come into use for research, there should be a recognition that evidence of calibration is required before publication. The solution to most of the limitations of these low-cost approaches is to develop a stronger community for sharing protocols, for sensor development, calibration and data processing. Initiatives such as PublicLab, targeting environmental research, is a good example for the phenotyping community looking at developing new technologies in-house. As discussed in Section 6, know-how sharing initiatives and online platforms should provide an online library of protocols and DIY instrumentation to help others to avoid ‘reinventing the wheel’. This co-operation should provide the right mechanisms for producing high-quality phenotyping information that is comparable across multiple experiments and environments.

## 4 Data processing and handling

Phenotyping complex traits demands the integration of data on different morphological, physiological and environmental variables [7,72]. Further, there is a need for data with higher temporal and spatial resolution for the characterization of the dynamic responses of plant function to the fluctuations of the environment. Consequently, the plant phenotyping platforms are requesting an increasing number of sensors to generate more complex datasets in an automated mode. This has generated an unprecedented, massive amount of data that normally exceeds our capacity to analyse it. Research groups often underestimate the resource required to store and manipulate terabytes of data. Efforts to optimize the automation of the data management and processing are usually overlooked, thus it has become a bottleneck within the HTP pipeline.

Measurement data can be classified into non-imaging and imaging, according to the kind of sensor used. Non-imaging data correspond to an integrated measurement over the area covered by the sensor. Thus, they usually correspond to a single value per variable (except in multi- or hyperspectral sensors where there is one value per each waveband), which makes the size of the data manageable. This is an advantage when trying to integrate time-series measurements from different sensors to support fast decision making in platforms for plant breeding. Nevertheless, such a system relies on fast data transfer and sensor synchronization (usually through a wireless network), technologies that are actively under development [[73], [74], [75]].

Imaging data are a spatial representation of a variable measured by each pixel in the detector array. Imaging sensors provide the opportunity to obtain spatial and temporal information of plant traits while reducing the acquisition time and errors associated with the data collection. However, the size and complexity of the data generated by such sensors greatly undermine the possibility to use them in HTP platforms. While in greenhouses images are usually transferred in real time to a server, the possibility of doing so becomes more limited in the field, especially using aerial platforms where the amount of data collected can be in the order of gigabytes, and therefore is limited by the on-board storage capacity. In some rural settings, sufficient network signal strength can be problematic. The retrieval of meaningful data from imaging sensors usually involves some degree of pre-processing. Among them we can find: calibrations, geo-referencing, orthorectification, pattern recognition, 3D reconstruction and machine learning [[36], [37], [38], [39]]. Images collected from aerial platforms require geometric and radiometric correction. The automation and speed of these steps depends on the image quality, the complexity of the algorithms used and the available computing power. Advances in this matter have been achieved for screening in batch mode structural traits such plant height, leaf area, biovolume and leaf angles of thousands of plants per day under greenhouse conditions [[76], [77], [78]]. Under field conditions, the challenge is the automation of the aerial data processing. Few software are available that automate image calibration and correction, obtaining good field maps of the studied variable. Furthermore, new developments aim to do real-time processing on-board in aerial platforms, delivering almost instantaneous maps of spectral indices such as NDVI. Despite these advances, there are intermediate steps that require some level of manual interaction, which slow the process, such as the identification of geodetic ground control points for geo-referencing, defining individual plot boundaries and retrieving plot-level data. Computing power can be a limiting factor in processing image data. For small to medium datasets, working with local desktop PCs are sufficiently fast to access and analyse the data. However, as the size and amount of data grow, this option can become expensive; thus, for big data cloud processing becomes an excellent option, which can increase computing power and storage capacity. There are an increasing number of cloud service providers, ensuring accessible prices and flexibility to process data.

## 5 Harmonising data

Phenotyping techniques developed during recent years have permitted the massive acquisition of datasets containing information originating from different sensors (e.g. RGB and hyperspectral cameras, see Table 1) at different scales (e.g. field and controlled conditions) and levels of organisation (e.g. canopy and leaf level). These datasets are unique resources, containing insightful information on a number of traits describing plant function and form (see Table 1). If made available to scientific community, these datasets can be further mined or combined in meta-analyses to generate new insight; for example on crop adaptation to multiple stresses and to accelerate breeding [2,79]. However, making them available is a major challenge for the plant phenomics community.

The first problem arises from the necessity to handle the huge amount of data generated by phenotyping facilities and devices. Whereas most informatics solutions accompanying high-throughput techniques have focused on the development of specific image analysis tools [80,81], little attention has focused on the development of information systems to handle, integrate and analyse the massive amount of sensor-derived data, with the added complexity of its heterogeneous nature originating from multiple sources [82]. This complexity is the challenge of big data [83,84], which can be described by: (i) the Volume, given the exponential increase of data acquired by phenotyping techniques at high spatial and temporal resolution; (ii) the Variety of data due to the multiplicity of data sources, the growing availability of sensors, and the need for the integration of metadata and knowledge (e.g. annotated data in lab books, protocols, manual measurements); (iii) the Velocity, given by the necessity to provide scientists with fast and powerful visualisation and analysis tools to inspect and handle the large amounts of experimental data acquired and analysed in real time; (iv) the Value, as phenomic experiments are expensive and nearly impossible to reproduce exactly (especially under field conditions) because of differences in environmental conditions; and (v) the Veracity, related to the necessity to track provenance data such as the successive steps, calibration of sensors, parameter settings, and methods that have been used to produce a given result.

The second problem is related to the necessity to enable interoperability between datasets and infrastructures, and issues surrounding Open Data are being actively discussed internationally. Initiatives such as the IAA (International Agroinformatics Alliance), a coalition of public and private institutions hosted at the supercomputing facility at the University of Minnesota, USA [85], and the farmer-oriented Open Ag Data Alliance (openag.io) are steps in this direction, enabling interoperability of data, while also respecting security and privacy. Similarly, a number of initiatives in the context of European plant phenotyping (EPPN^2020^ (http://eppn2020.plant-phenotyping.eu/), Trans-PLANT (http://transplantdb.eu/), ELIXIR-EXCELERATE (https://www.elixir-europe.org/excelerate/plants), EMPHASIS (http://emphasis.plant-phenotyping.eu/), among others) have tackled these issues by developing standardized protocols [83,86,87] and software frameworks for phenotyping experiments [82,88] following the FAIR data principles (Findable, Accessible, Interoperable and Reusable) [89]. These initiatives, such as MIAPPE (http://www.miappe.org/) and the ISA-Tab framework [81] (isa-tools.org) have established a list of attributes to fully describe phenotyping experiments and comprehensive metadata descriptions using standards and ontologies. More recently, the ontology-driven PHIS Information System (www.phis.inra.fr) has been proposed as an open-source solution for integrating, managing and sharing multi-source and multi-scale data in plant phenomics experiments for both controlled and field conditions [82]. Ontologies are powerful tools for formalising the different relationships established between the different objects involved in phenotyping experiments (e.g. plants, plots, sensors), and to enable the interconnection with other available biological databases and resources [90,91]. The Planteome project (http://www.planteome.org) gathers a suite of reference and species-specific ontologies [92], including the Crop Ontology (http://cropontology.org/) [93] and Plant Ontology (http://plantontology.org/) [94]), which provide relevant terms and concept hierarchies related to the anatomy, structure and phenotype of crops. However, because plant phenotyping is by essence multi-source and multi-scale, new ontologies are needed to fulfil the necessary concepts in phenotyping experiments, and thus to enable full interoperability between datasets. It is recognized that many alternative terminologies for traits exist in local usage, and while these may never be replaced by official Ontological terms for everyday use, they can easily translate to and map onto accepted ontologies when data are uploaded into a database. Taken together, these new opportunities in data management may help the assembly of datasets originating from multiple sources, resulting in unprecedented amount of information that can be re-used, combined and re-analysed to generate new knowledge.

## 6 Sharing know-how

Websites and online consultable databases with methods and protocols have been established to share phenotyping know-how. A major objective of the EU-funded DROPS project was to train scientists and disseminate know-how in the use of up-to-date methods of plant measurement and principles of breeding for tolerance to water deficit via training courses and a website (https://www6.inra.fr/dropsproject/). The CGIAR Centres regularly host training phenotyping workshops for researchers around the world. Another helpful resource is a crowd-sourced database to access and share protocols in plant eco-physiology (http://prometheuswiki.org). The Index Database collates information on remote sensing indices, sensors and applications (http://www.indexdatabase.de/). Online courses and webinars such as the ‘Plant Phenomics Phridays’ series (https://bigdata.unl.edu/fall-seminar-series-plant-phenomics-phridays) help share the latest developments. To foster development of computer vision for phenotyping, an expanding image database is available (plant-phenotyping.org) and the plant-image-analysis.org database (http://www.plant-image-analysis.org) provides and extensive and curated list of existing plant image analysis software tools for users and developers [95]. There is a need for greater international co-ordination and centralized collation of methods, and perhaps a publishing house may wish to host a website for phenotyping protocols. Transferring methodological and technological progress from research to operative breeding is one goal of the community. However, its adoptability will rely on making methods and instruments simple, fast, inexpensive, and demonstrating that phenotyping can increase genetic gain in breeding programmes.

## 7 Conclusion

While substantial advances have been made in molecular breeding techniques, phenotyping throughput and costs remain the bottleneck to breeding progress. Further advancement in high-throughput screening is essential to take full advantage of genomic resources to dissect the genetic control of quantitative traits, particularly those related to yield components and stress tolerance.

High-throughput phenotyping technologies have been originally developed for the greenhouse and the development of technologies for field applications lacking behind. While environmentally controlled conditions allow high precision measurements, field phenotyping is posing various different challenges and limitations due to the determination of traits in a multifactorial environment. This classical dichotomy is currently further expanded by establishing phenotyping facilities with field like growth conditions in combination with extensive environmental control.

Many of the issues we discuss in the plant phenotyping community have been, and are continuing to be discussed in other sectors, and there can be benefit in learning how others approach these challenges, for instance in murine phenotyping [96]. Genotypic data are now handled and shared in ways that set a precedent for phenotypic data, although connecting genomic and phenomic datasets remains challenging [97]. Integration of these data into simulation models to predict trait value is of increasing importance [2,98]. We can envision that eventually genotypic, phenotypic, environmental and agronomic/plant management data will be harmonised on an international scale, but significant hurdles remain before that is achieved. Along the way, it is vital that the accessibility, integrity and veracity of the data that are being collected are maintained so that they retain value over time. Calibration of sensors, particularly low-cost or DIY sensors is of particular importance. Universal standardisation of experimental protocols may not be achieved quickly, but transparent quality control, documentation according to minimum reporting standards and ways to store annotation and metadata are required.

The availability of appropriate and novel sensors is key to realize urgent technological development needs. Likewise, innovations in specialized solutions for image pre-processing, analyses and data management are needed. Development of sophisticated or low cost, user-friendly sensor solutions for specialized uses will require close interactions across disciplines and active engagement of engineers and manufacturers. Sensor and image data need to be integrated with other multi-omics data to create a holistic, second generation phenomics approach.

## References

[1] Araus J.L., Cairns J.E. (2014). Field high-throughput phenotyping: the new crop breeding frontier. Trends Plant Sci..

[2] Tardieu F., Cabrera-Bosquet L., Pridmore T., Bennett M., Phenomics Plant (2017). From sensors to knowledge. Curr. Biol..

[3] Fiorani F., Schurr U., Merchant S.S. (2013). Future scenarios for plant phenotyping.

[4] Coppens F., Wuyts N., Inzé D., Dhondt S. (2017). Unlocking the potential of plant phenotyping data through integration and data-driven approaches. Curr. Opin. Syst. Biol..

[5] Pauli D., Chapman S.C., Bart R., Topp C.N., Lawrence-Dill C.J., Poland J., Gore M.A. (2016). The quest for understanding phenotypic variation via integrated approaches in the field environment. Plant Physiol..

[6] Li J., Rascher U., Müller-Linow M., Cendrero-Mateo M.P., Pieruschka R., Albrecht H., Pinto F., Gatzke S., Rischbeck P., Keller B. (2017). Field phenotyping: concepts and examples to quantify dynamic plant traits across scales in the Field. Terrestrial Ecosystem Research Infrastructures.

[7] (2016). 4th International Plant Phenotyping Symposium, Mexico4th International Plant Phenotyping Symposium2016. 4th International Plant Phenotyping Symposium.

[8] Großkinsky D.K., Jaya Syaifullah S., Roitsch T., Lim P. (2017). Integration of multi-omics techniques and physiological phenotyping within a holistic phenomics approach to study senescence in model and crop plants. J. Exp. Bot..

[9] Moore G.K. (1979). What is a picture worth? A history of remote sensing/Quelle est la valeur d’une image? Un tour d’horizon de télédétection. Hydrol. Sci. Bull..

[10] Rebetzke G.J., Jimenez-Berni J.A., Bovill W.D., Deery D.M., James R.A. (2016). High-throughput phenotyping technologies allow accurate selection of stay-green. J. Exp. Bot..

[11] Jimenez-Berni J.A., Deery D.M., Rozas-Larraondo P., Condon A.T.G., Rebetzke G.J., James R.A., Bovill W.D., Furbank R.T., Sirault X.R. (2018). High throughput determination of plant height, ground cover, and above-ground biomass in wheat with LiDAR. Front. Plant Sci..

[12] Eitel J.U., Magney T.S., Vierling L.A., Brown T.T., Huggins D.R. (2014). LiDAR based biomass and crop nitrogen estimates for rapid, non-destructive assessment of wheat nitrogen status. Field Crops Res..

[13] Geipel J., Link J., Claupein W. (2014). Combined spectral and spatial modeling of corn yield based on aerial images and crop surface models acquired with an unmanned aircraft system. Remote Sens..

[14] Tilly N., Hoffmeister D., Cao Q., Lenz-Wiedemann V., Miao Y., Bareth G. (2015). Transferability of models for estimating paddy rice biomass from spatial plant height data. Agriculture.

[15] Kaasalainen S., Holopainen M., Karjalainen M., Vastaranta M., Kankare V., Karila K., Osmanoglu B. (2015). Combining lidar and synthetic aperture radar data to estimate forest biomass: status and prospects. Forests.

[16] J.G. Harris, A. Krishnaswamy, S. Byer, Method and system for dynamic, luminance-based color contrasting in a region of interest in a graphic image in, Google Patents, 2011.

[17] Dai W., Yang B., Dong Z., Shaker A. (2018). A new method for 3D individual tree extraction using multispectral airborne LiDAR point clouds. ISPRS J. Photogramm. Remote. Sens..

[18] Danson F.M., Gaulton R., Armitage R.P., Disney M., Gunawan O., Lewis P., Pearson G., Ramirez A.F. (2014). Developing a dual-wavelength full-waveform terrestrial laser scanner to characterize forest canopy structure. Agric. For. Meteorol..

[19] Douglas E., Martel J., Cook T., Mendill C., Marshall R., Chakrabarti S., Strahler A., Schaaf C., Woodcock C., Liu Z. (2012). A dual-wavelength echidna lidar for Ground-based Forest scanning. Proceedings of SilviLaser.

[20] Wallace A.M., McCarthy A., Nichol C.J., Ren X., Morak S., Martinez-Ramirez D., Woodhouse I.H., Buller G.S. (2014). Design and evaluation of multispectral lidar for the recovery of arboreal parameters. IEEE Trans. Geosci. Remote. Sens..

[21] Wei G., Shalei S., Bo Z., Shuo S., Faquan L., Xuewu C. (2012). Multi-wavelength canopy LiDAR for remote sensing of vegetation: design and system performance. ISPRS J. Photogramm. Remote. Sens..

[22] Morsy S., Shaker A., El-Rabbany A. (2017). Multispectral LiDAR data for land cover classification of urban areas. Sensors.

[23] van Rees E. (2015). The first multispectral airborne lidar sensor. GeoInformatics.

[24] Vadez V., Kholova J., Hummel G., Zhokhavets U., Gupta S.K., Hash C.T. (2015). LeasyScan: a novel concept combining 3D imaging and lysimetry for high-throughput phenotyping of traits controlling plant water budget. J. Exp. Bot..

[25] Kuhlgert S., Austic G., Zegarac R., Osei-Bonsu I., Hoh D., Chilvers M.I., Roth M.G., Bi K., TerAvest D., Weebadde P. (2016). MultispeQ Beta: a tool for large-scale plant phenotyping connected to the open PhotosynQ network. R. Soc. Open Sci..

[26] Kolber Z., Klimov D., Ananyev G., Rascher U., Berry J., Osmond B. (2005). Measuring photosynthetic parameters at a distance: laser induced fluorescence transient (LIFT) method for remote measurements of photosynthesis in terrestrial vegetation. Photosynthesis Res..

[27] Raesch A., Muller O., Pieruschka R., Rascher U. (2014). Field observations with laser-induced fluorescence transient (LIFT) method in barley and sugar beet. Agriculture.

[28] Pinto F., Damm A., Schickling A., Panigada C., Cogliati S., Müller‐Linow M., Balvora A., Rascher U. (2016). Sun‐induced chlorophyll fluorescence from high‐resolution imaging spectroscopy data to quantify spatio‐temporal patterns of photosynthetic function in crop canopies. Plant Cell Environ..

[29] Deery D.M., Rebetzke G.J., Jimenez-Berni J.A., James R.A., Condon A.G., Bovill W.D., Hutchinson P., Scarrow J., Davy R., Furbank R.T. (2016). Methodology for high-throughput field phenotyping of canopy temperature using airborne thermography. Front. Plant Sci..

[30] Chapman S., Merz T., Chan A., Jackway P., Hrabar S., Dreccer M., Holland E., Zheng B., Ling T., Jimenez-Berni J. (2014). Pheno-Copter: a low-altitude, autonomous remote-sensing robotic helicopter for high-throughput field-based phenotyping. Agronomy.

[31] Camino C., González-Dugo V., Hernández P., Sillero J.C., Zarco‐Tejada P.J. (2018). Improved nitrogen retrievals with airborne-derived fluorescence and plant traits quantified from VNIR-SWIR hyperspectral imagery in the context of precision agriculture. Int. J. Appl. Earth Obs. Geoinf..

[32] Yang G., Liu J., Zhao C., Li Z., Huang Y., Yu H., Xu B., Yang X., Zhu D., Zhang X., Zhang R., Feng H., Zhao X., Li Z., Li H., Yang H. (2017). Unmanned aerial vehicle remote sensing for field-based crop phenotyping: current status and perspectives. Front. Plant Sci..

[33] Underwood J., Wendel A., Schofield B., McMurray L., Kimber R. (2017). Efficient in-field plant phenomics for row-crops with an autonomous ground vehicle. J. Field Robot..

[34] Wendel A., Underwood J. (2017). Illumination compensation in ground based hyperspectral imaging. ISPRS J. Photogramm. Remote. Sens..

[35] Gonzalez-Dugo V., Hernandez P., Solis I., Zarco-Tejada P. (2015). Using high-resolution hyperspectral and thermal airborne imagery to assess physiological condition in the context of wheat phenotyping. Remote Sens..

[36] Ngo Q.M., Ho Y.-L.D., Pugh J.R., Sarua A., Cryan M.J. (2018). Enhanced UV/blue fluorescent sensing using metal-dielectric-metal aperture nanoantenna arrays. Curr. Appl. Phys..

[37] Dorh N., Sarua A., Stokes J., Hueting N., Cryan M. (2016). Fluorescent emission enhancement by aluminium nanoantenna arrays in the near UV. J. Opt..

[38] Valle B., Simonneau T., Boulord R., Sourd F., Frisson T., Ryckewaert M., Hamard P., Brichet N., Dauzat M., Christophe A. (2017). PYM: a new, affordable, image-based method using a Raspberry Pi to phenotype plant leaf area in a wide diversity of environments. Plant Methods.

[39] Zhou B., Elazab A., Bort J., Vergara O., Serret M.D., Araus J.L. (2015). Low-cost assessment of wheat resistance to yellow rust through conventional RGB images. Comput. Electron. Agric..

[40] Yu K., Kirchgessner N., Grieder C., Walter A., Hund A. (2017). An image analysis pipeline for automated classification of imaging light conditions and for quantification of wheat canopy cover time series in field phenotyping. Plant Methods.

[41] Ghamkhar K., Irie K., Hagedorn M., Hsiao J., Fourie J., Gebbie S., Flay C., Barrett B., Stewart A., Werner A. (2018). Using LIDAR for forage yield measurement of perennial ryegrass (Lolium perenne L.) field plots. Breeding Grasses and Protein Crops in the Era of Genomics.

[42] Siebers M., Edwards E., Jimenez-Berni J., Thomas M., Salim M., Walker R. (2018). Fast phenomics in vineyards: development of GRover, the grapevine rover, and LiDAR for assessing grapevine traits in the field. Sensors.

[43] Hu P., Chapman S.C., Wang X., Potgieter A., Duan T., Jordan D., Guo Y., Zheng B. (2018). Estimation of plant height using a high throughput phenotyping platform based on unmanned aerial vehicle and self-calibration: example for sorghum breeding. Eur. J. Agron..

[44] Shi Y., Thomasson J.A., Murray S.C., Pugh N.A., Rooney W.L., Shafian S., Rajan N., Rouze G., Morgan C.L., Neely H.L. (2016). Unmanned aerial vehicles for high-throughput phenotyping and agronomic research. PLoS One.

[45] Holmgren J., Nilsson M., Olsson H. (2003). Estimation of tree height and stem volume on plots using airborne laser scanning. For. Sci..

[46] Lefsky M.A., Cohen W., Acker S., Parker G.G., Spies T., Harding D. (1999). Lidar remote sensing of the canopy structure and biophysical properties of Douglas-fir western hemlock forests. Remote Sens. Environ..

[47] Næsset E. (2002). Predicting forest stand characteristics with airborne scanning laser using a practical two-stage procedure and field data. Remote Sens. Environ..

[48] Ehlert D., Adamek R., Horn H.-J. (2009). Laser rangefinder-based measuring of crop biomass under field conditions. Precis. Agric..

[49] Harding D., Lefsky M., Parker G., Blair J. (2001). Laser altimeter canopy height profiles: methods and validation for closed-canopy, broadleaf forests. Remote Sens. Environ..

[50] Lovell J., Jupp D.L., Culvenor D., Coops N. (2003). Using airborne and ground-based ranging lidar to measure canopy structure in Australian forests. Can. J. Remote. Sens..

[51] Saeys W., Lenaerts B., Craessaerts G., De Baerdemaeker J. (2009). Estimation of the crop density of small grains using LiDAR sensors. Biosyst. Eng..

[52] Barrett B., Faville M.J., Ghamkhar K., Carena M.J. (2018). Developing new tools for pasture plant breeding. J. N. Z. Grasslands.

[53] Mairhofer S., Zappala S., Tracy S.R., Sturrock C., Bennett M., Mooney S.J., Pridmore T. (2012). RooTrak: automated recovery of three-dimensional plant root architecture in soil from X-ray Micro-Computed Tomography Images using visual tracking. Plant Physiol..

[54] Jeudy C., Adrian M., Baussard C., Bernard C., Bernaud E., Bourion V., Busset H., Cabrera-Bosquet L., Cointault F., Han S.M., Lamboeuf M., Moreau D., Pivato B., Prudent M., Trouvelot S., Truong H.N., Vernoud V., Voisin A.S., Wipf D., Salon C. (2016). RhizoTubes as a new tool for high throughput imaging of plant root development and architecture: test, comparison with pot grown plants and validation. Plant Methods.

[55] Whalley W.R., Binley A., Watts C., Shanahan P., Dodd I.C., Ober E., Ashton R., Webster C., White R., Hawkesford M.J. (2017). Methods to estimate changes in soil water for phenotyping root activity in the field. Plant Soil.

[56] Liu X., Dong X., Xue Q., Leskovar D.I., Jifon J., Butnor J.R., Marek T. (2017). Ground penetrating radar (GPR) detects fine roots of agricultural crops in the field. Plant Soil.

[57] Delgado A., Hays D.B., Bruton R.K., Ceballos H., Novo A., Boi E., Selvaraj M.G. (2017). Ground penetrating radar: a case study for estimating root bulking rate in cassava (*Manihot esculenta* Crantz). Plant Methods.

[58] Jarrahi M. (2015). Advanced photoconductive terahertz optoelectronics based on nano-antennas and nano-plasmonic light concentrators. IEEE Trans. Terahertz Sci. Technol..

[59] Breitenstein B., Scheller M., Shakfa M.K., Kinder T., Müller-Wirts T., Koch M., Selmar D. (2011). Introducing terahertz technology into plant biology: a novel method to monitor changes in leaf water status. J. Appl. Bot. Food Qual..

[60] Born N., Behringer D., Liepelt S., Beyer S., Schwerdtfeger M., Ziegenhagen B., Koch M. (2014). Monitoring plant drought stress response using terahertz time-domain spectroscopy. Plant Physiol..

[61] Gente R., Koch M. (2015). Monitoring leaf water content with THz and sub-THz waves. Plant Methods.

[62] Krügener K., Busch S., Soltani A., Schwerdtfeger M., Castro-Camus E., Koch M., Viöl W. (2017). THz time domain spectroscopy—Non-destructive evaluation of material detachments from exposed natural stone and ceramic objects. Infrared, Millimeter, and Terahertz Waves (IRMMW-THz), 2017 42nd International Conference on, IEEE.

[63] Holl P.M., Reinhard F. (2017). Holography of wi-fi radiation. Phys. Rev. Lett..

[64] Jones H.G., Hutchinson P.A., May T., Jamali H., Deery D.M. (2018). A practical method using a network of fixed infrared sensors for estimating crop canopy conductance and evaporation rate. Biosys. Eng..

[65] Berni J., Zarco-Tejada P., Sepulcre-Cantó G., Fereres E., Villalobos F. (2009). Mapping canopy conductance and CWSI in olive orchards using high resolution thermal remote sensing imagery. Remote Sens. Environ..

[66] Zarco-Tejada P.J., González-Dugo V., Berni J.A. (2012). Fluorescence, temperature and narrow-band indices acquired from a UAV platform for water stress detection using a micro-hyperspectral imager and a thermal camera. Remote Sens. Environ..

[67] Paulus S., Behmann J., Mahlein A.-K., Plümer L., Kuhlmann H. (2014). Low-cost 3D systems: suitable tools for plant phenotyping. Sensors.

[68] Barmeier G., Mistele B., Schmidhalter U. (2017). Referencing laser and ultrasonic height measurements of barleycultivars by using a herbometre as standard. Crop Pasture Sci..

[69] Dobrescu A., Scorza L.C., Tsaftaris S.A., McCormick A.J. (2017). A “Do-It-Yourself” phenotyping system: measuring growth and morphology throughout the diel cycle in rosette shaped plants. Plant Methods.

[70] Reynolds D., Baret F., Welcker C., Bostrom A., Ball J., Cellini F., Lorence A., Chawade A., Khafif M., Noshita K., Mueller-Linow M., Zhou J., Tardieu F. (2019). What is cost-efficient phenotyping? Optimizing costs for different scenarios. Plant Sci..

[71] Yokogawa S., Oshiyama I., Ikeda H., Ebiko Y., Hirano T., Saito S., Oinoue T., Hagimoto Y., Iwamoto H. (2017). IR sensitivity enhancement of CMOS Image Sensor with diffractive light trapping pixels. Sci. Rep..

[72] Großkinsky D.K., Svensgaard J., Christensen S., Roitsch T. (2015). Plant phenomics and the need for physiological phenotyping across scales to narrow the genotype-to-phenotype knowledge gap. J. Exp. Bot..

[73] Salehi A., Jimenez-Berni J., Deery D.M., Palmer D., Holland E., Rozas-Larraondo P., Chapman S.C., Georgakopoulos D., Furbank R.T. (2015). SensorDB: a virtual laboratory for the integration, visualization and analysis of varied biological sensor data. Plant Methods.

[74] Fukatsu T., Hirafuji M. (2005). Field monitoring using sensor-nodes with a web server. JRM.

[75] Utsushi H., Abe A., Tamiru M., Ogasawara Y., Obara T., Sato E., Ochiai Y., Terauchi R., Takagi H. (2015). WIPPER: an accurate and efficient field phenotyping platform for large-scale applications. Breed. Sci..

[76] Brichet N., Fournier C., Turc O., Strauss O., Artzet S., Pradal C., Welcker C., Tardieu F., Cabrera-Bosquet L. (2017). A robot-assisted imaging pipeline for tracking the growths of maize ear and silks in a high-throughput phenotyping platform. Plant Methods.

[77] Cabrera-Bosquet L., Fournier C., Brichet N., Welcker C., Suard B., Tardieu F. (2016). High-throughput estimation of incident light, light interception and radiation-use efficiency of thousands of plants in a phenotyping platform. New Phytol..

[78] Chen T.W., Cabrera-Bosquet L., Alvarez Prado S., Perez R., Artzet S., Pradal C., Coupel-Ledru A., Fournier C., Tardieu F. (2018). Genetic and environmental dissection of biomass accumulation in multi-genotype maize canopies. J. Exp. Bot..

[79] Adam-Blondon A.F., Alaux M., Pommier C., Cantu D., Cheng Z.M., Cramer G.R., Davies C., Delrot S., Deluc L., Di Gaspero G., Grimplet J., Fennell A., Londo J.P., Kersey P., Mattivi F., Naithani S., Neveu P., Nikolski M., Pezzotti M., Reisch B.I., Töpfer R., Vivier M.A., Ware D., Quesneville H. (2016). Towards an open grapevine information system. Hortic. Res..

[80] Hartmann A., Czauderna T., Hoffmann R., Stein N., Schreiber F. (2011). HTPheno: an image analysis pipeline for high-throughput plant phenotyping. BMC Bioinformatics.

[81] Klukas C., Chen D., Pape J.-M. (2014). Integrated analysis platform: an open-source information system for high-throughput plant phenotyping. Plant Physiol..

[82] Neveu P., Tireau A., Hilgert N., Nègre V., Mineau-Cesari J., Brichet N., Chapuis R., Sanchez I., Pommier C., Charnomordic B., Tardieu F., Cabrera-Bosquet L. (2019). Dealing with multi-source and multi-scale information in plant phenomics: the ontology-driven Phenotyping Hybrid Information System. New Phytol..

[83] Krajewski P., Chen D., Ćwiek H., van Dijk A.D.J., Fiorani F., Kersey P., Klukas C., Lange M., Markiewicz A., Nap J.P., van Oeveren J., Pommier C., Scholz U., van Schriek M., Usadel B., Weise S. (2015). Towards recommendations for metadata and data handling in plant phenotyping. J. Exp. Bot..

[84] Higdon R., Haynes W., Stanberry L., Stewart E., Yandl G., Howard C., Broomall W., Kolker N., Kolker E. (2013). Unraveling the complexities of life sciences data. Big Data.

[85] Gustafson A., Erdmann J., Milligan M., Onsongo G., Pardey P., Prather T., Silverstein K., Wilgenbusch J., Zhang Y. (2017). A platform for computationally advanced collaborative agroInformatics data discovery and analysis. Proceedings of the Practice and Experience in Advanced Research Computing 2017 on Sustainability, Success and Impact.

[86] Junker A., Muraya M.M., Weigelt-Fischer K., Arana-Ceballos F., Klukas C., Melchinger A.E., Meyer R.C., Riewe D., Altmann T. (2014). Optimizing experimental procedures for quantitative evaluation of crop plant performance in high throughput phenotyping systems. Front. Plant Sci..

[87] Ćwiek-Kupczyńska H., Altmann T., Arend D., Arnaud E., Chen D., Cornut G., Fiorani F., Frohmberg W., Junker A., Klukas C., Lange M., Mazurek C., Nafissi A., Neveu P., van Oeveren J., Pommier C., Poorter H., Rocca-Serra P., Sansone S.-A., Scholz U., van Schriek M., Seren Ü., Usadel B., Weise S., Kersey P., Krajewski P. (2016). Measures for interoperability of phenotypic data: minimum information requirements and formatting. Plant Methods.

[88] Arend D., Lange M., Chen J., Colmsee C., Flemming S., Hecht D., Scholz U. (2014). e!DAL--a framework to store, share and publish research data. BMC Bioinformatics.

[89] Wilkinson M.D., Dumontier M., Aalbersberg I.J., Appleton G., Axton M., Baak A., Blomberg N., Boiten J.-W., da Silva Santos L.B., Bourne P.E., Bouwman J., Brookes A.J., Clark T., Crosas M., Dillo I., Dumon O., Edmunds S., Evelo C.T., Finkers R., Gonzalez-Beltran A., Gray A.J.G., Groth P., Goble C., Grethe J.S., Heringa J., ’t Hoen P.A.C., Hooft R., Kuhn T., Kok R., Kok J., Lusher S.J., Martone M.E., Mons A., Packer A.L., Persson B., Rocca-Serra P., Roos M., van Schaik R., Sansone S.-A., Schultes E., Sengstag T., Slater T., Strawn G., Swertz M.A., Thompson M., van der Lei J., van Mulligen E., Velterop J., Waagmeester A., Wittenburg P., Wolstencroft K., Zhao J., Mons B. (2016). The FAIR Guiding Principles for scientific data management and stewardship. Sci. Data.

[90] Mungall C.J., Gkoutos G.V., Smith C.L., Haendel M.A., Lewis S.E., Ashburner M. (2010). Integrating phenotype ontologies across multiple species. Genome Biol..

[91] Robinson P.N., Webber C. (2014). Phenotype ontologies and cross-species analysis for translational research. PLoS Genet..

[92] Cooper L., Meier A., Laporte M.A., Elser J.L., Mungall C., Sinn B.T., Cavaliere D., Carbon S., Dunn N.A., Smith B., Qu B., Preece J., Zhang E., Todorovic S., Gkoutos G., Doonan J.H., Stevenson D.W., Arnaud E., Jaiswal P. (2018). The Planteome database: an integrated resource for reference ontologies, plant genomics and phenomics. Nucleic Acids Res..

[93] Shrestha R., Matteis L., Skofic M., Portugal A., McLaren G., Hyman G., Arnaud E. (2012). Bridging the phenotypic and genetic data useful for integrated breeding through a data annotation using the Crop Ontology developed by the crop communities of practice. Front. Physiol..

[94] Ilic K., Kellogg E.A., Jaiswal P., Zapata F., Stevens P.F., Vincent L.P., Avraham S., Reiser L., Pujar A., Sachs M.M., Whitman N.T., McCouch S.R., Schaeffer M.L., Ware D.H., Stein L.D., Rhee S.Y. (2007). The plant structure ontology, a unified vocabulary of anatomy and morphology of a flowering plant. Plant Physiol..

[95] Lobet G., Draye X., Périlleux C. (2013). An online database for plant image analysis software tools. Plant Methods.

[96] Maier H., Leuchtenberger S., Fuchs H., Gailus-Durner V., de Angelis M.H. (2017). Big data in large-scale systemic mouse phenotyping. Curr. Opin. Syst. Biol..

[97] Bolger M., Schwacke R., Gundlach H., Schmutzer T., Chen J., Arend D., Oppermann M., Weise S., Lange M., Fiorani F. (2017). From plant genomes to phenotypes. J. Biotechnol..

[98] Brown T.B., Cheng R., Sirault X.R.R., Rungrat T., Murray K.D., Trtilek M., Furbank R.T., Badger M., Pogson B.J., Borevitz J.O. (2014). TraitCapture: genomic and environment modelling of plant phenomic data. Curr. Opin. Plant Biol..

